# Mechanisms of Pathogenic *Candida* Species to Evade the Host Complement Attack

**DOI:** 10.3389/fcimb.2020.00094

**Published:** 2020-03-12

**Authors:** Dhirendra Kumar Singh, Renáta Tóth, Attila Gácser

**Affiliations:** ^1^Department of Microbiology, University of Szeged, Szeged, Hungary; ^2^MTA-SZTE Lendület Mycobiome Research Group, University of Szeged, Szeged, Hungary

**Keywords:** *Candida*, secreted proteases, complement system, fungal infection, innate immune response, pathogenesis

## Abstract

*Candida* species are common colonizers of the human skin, vagina, and the gut. As human commensals, *Candida* species do not cause any notable damage in healthy individuals; however, in certain conditions they can initiate a wide range of diseases such as chronic disseminated candidiasis, endocarditis, vaginitis, meningitis, and endophthalmitis. The incidence of *Candida* caused infections has increased worldwide, with mortality rates exceeding 70% in certain patient populations. *C. albicans, C. glabrata, C. tropicalis, C. parapsilosis*, and *C. krusei* are responsible for more than 90% of *Candida*-related infections. Interestingly, the host immune response against these closely related fungi varies. As part of the innate immune system, complement proteins play a crucial role in host defense, protecting the host by lysing pathogens or by increasing their phagocytosis by phagocytes through opsonization. This review summarizes interactions of host complement proteins with pathogenic *Candida* species, including *C. albicans* and non*-albicans Candida* species such as *C. parapsilosis*. We will also highlight the various ways of complement activation, describe the antifungal effects of complement cascades and explore the mechanisms adopted by members of pathogenic *Candida* species for evading complement attack.

## Infections Caused by *Candida* Species

*Candida* species are common colonizers of various mucosal surfaces, including that of the oral cavity, gut or vagina; however, in the setting of certain predisposing conditions they are able to disseminate throughout the host. The increasing incidence of invasive fungal diseases is a global phenomenon (Park et al., 2009; Thomas et al., 2010; Vallabhaneni et al., 2016; Tóth et al., 2019).

*C. albicans*, as the most common cause of candidiasis, is studied more extensively than any other *Candida* species. Nonetheless, increasing incidence of candidemia caused by non- *albicans Candida* (NAC) species has also been reported in the latest decade, that led to the rise of NAC investigations (Andes et al., 2016; Strollo et al., 2016). Their potential to cause outbreaks, higher resistance to antifungal drugs, and the ability to cause recurrent infections has led to this higher scrutiny (Lee et al., 2018). According to the Centers for Disease Control and Prevention (CDC), ~25,000 cases of candidemia occur each year in the USA (Mehta et al., 2018). Current annual burden rates in the United Kingdom for invasive candidiasis is ~5,000 cases (Pegorie et al., 2017). For other countries in Europe, the incidences for invasive candidiasis have been reported as 3.9 /100,000 in Norway, 8.6/100,000 in Denmark, and 8.1/100,000 cases in Spain, which also has had a 1.88-fold increase in incidence in the last decade (Rodriguez-Tudela et al., 2015; Lamoth et al., 2018). The average incidence of candidemia in Australia is 2.4/100,000, whereas regionally the range varied from 1.6 to 7.2/100,000 population (Chapman et al., 2017). A recent review summarized data from 39 papers containing reports from across the globe and estimated a total of 159,253 candidemia episodes by August 2017, including a high prevalence in Pakistan followed by Brazil and Russia with the lowest incidence in Jamaica, Austria, and Portugal (Bongomin et al., 2017). Among NAC species, *C. parapsilosis, C. glabrata*, and *C. tropicalis* have been commonly associated with candidemia among cancer patients in the USA, Portugal and Australia (Sipsas et al., 2009; Pammi et al., 2013; Pfaller et al., 2014; Wu et al., 2017). In Asian countries, higher mortality rates are associated with NAC species (Ma et al., 2013; Pinhati et al., 2016). In general, cases of candidemia increased nearly 5-fold in the last 10 years, with the highest increase of 4–15-fold recorded in developing countries in which recurrent episodes were frequent (Kaur and Chakrabarti, 2017). Crude mortality rates among patients with invasive candidiasis or candidemia generally range between 40 to 60%, depending on the underlining conditions (Wu et al., 2017). Increasing incidences of candidemia have occurred in pediatric ICUs, particularly in developing countries in which there are limited resources, a dearth of advanced diagnostics, high patient loads, and a potential limited awareness about fungal diseases (Kaur and Chakrabarti, 2017). Given that *Candida* infections contribute to a relatively high morbidity and mortality, especially among patients admitted to ICUs, much attention has been paid on understanding the basics of their pathobiology, virulence factors, predisposing conditions along with the immune responses of both healthy and immune compromised individuals. Besides the cellular components of both the innate and adaptive immune system, the complement system has also been shown to play a fundamental role in fungal pathogen clearance, similarly to that of invading bacteria. Although the thick cell wall of pathogenic fungi builds a certain level of resistance to direct lysis due to complement activation, binding of complement factors to the fungal surface facilitates their phagocytosis and alters inflammatory responses from host immune cells (Kozel, 1996; Cheng et al., 2012; van Strijp et al., 2015). In the followings we summarize how various complement proteins shape defense mechanisms to prevent the development of disseminated candidiasis and how such mechanisms could be avoided by *Candida* species.

## Overview of the Complement Cascade

During infections, complement proteins facilitate the phagocytosis of invading pathogens by opsonization, initiate inflammatory responses and modify the behavior of B and T cells (Killick et al., 2017). The complement cascade is activated by three distinct routes.

**Figure 1:**
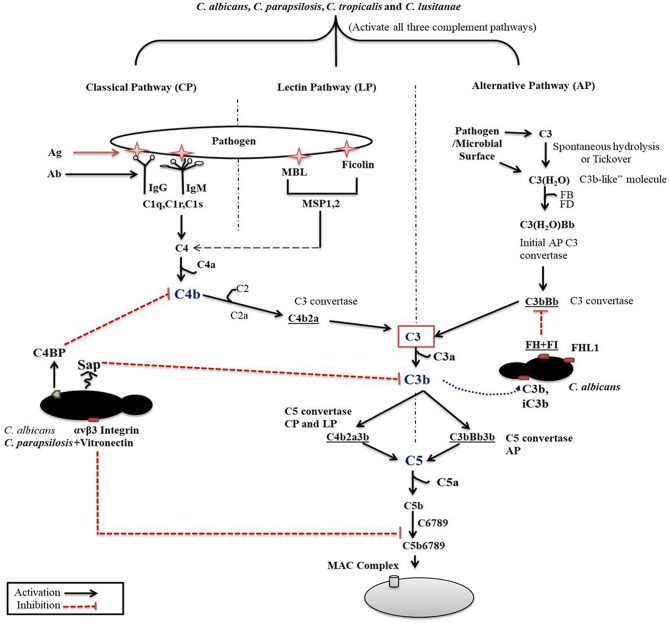
Complement cascade initiation after *Candida* recognition. The three distinct pathways of the complement cascade are referred to as classical, lectin and alternative. *C. albicans* can efficiently regulate complement cascades either by secreting aspartyl proteases or by binding with complement regulators on its surface.

The classical pathway (CP) is initiated by binding components of the C1 complex (C1q) with antigen bound IgM or IgG or by binding with other recognition molecules such as phosphatidylserine, C type reactive protein, pentraxins, serum amyloid P component, and various receptors including integrin α2β1 (Roy et al., 2017). C1q and antigen-carrier immunoglobulin binding ultimately leads to the activation C1s that cleaves C2 and C4 into C2a and C2b and C4a and C4b fragments, respectively. C4b then binds to cell surfaces and to C2a to form the C3 convertase (C4bC2a) (Figure 1). C3 convertase converts complement protein 3 (C3), the central component of the complement attack, into C3a (anaphylatoxin) and C3b (opsonin). Further attachment of C3b to the C4bC2a complex generates the C5 convertase. Binding of C3b to the surface of pathogenic species facilitates their phagocytosis (van Lookeren Campagne et al., 2007). C3b also undergoes internal cleavage to produce (inactive) iC3b and C3d. Both of these C3b fragments act as opsonins and further bind and label (opsonize) pathogens to facilitate phagocytosis (van Lookeren Campagne et al., 2007; Hostetter, 2008). Deposition of iC3b facilitates recognition by complement receptor 3 (CR3), that enhances phagocytosis, reactive oxygen species (ROS) production, leukocyte trafficking, and migration of macrophages and neutrophils (Hostetter, 2008; Netea et al., 2008). Macrophages in the kidney and liver are also involved in the clearance of pathogenic fungi (Lionakis et al., 2013; Coelho and Drummond, 2019; Sun et al., 2019). CR3 activation also leads to enhanced NET formation and pro- and anti-inflammatory cytokine production by both neutrophils and macrophages (Löfgren et al., 1999; Huang et al., 2015; Lukácsi et al., 2017). Proteolytic cleavage of surface-bound iC3b further creates the opsonizing fragment C3d and C3dg.

The previously mentioned C5 convertase, or C4bC2a(C3b)_n_, will initiate the formation of the membrane attack complex (MAC), thus the terminal pathway (Ali et al., 2012). C5 convertase cleaves C5, a terminal component of the complement cascade into C5a and C5b. C5a is an anaphylatoxin and a powerful mediator of inflammation, while C5b together with C6-C7-C8 and C9 is required for the formation of the terminal complement complex. C5a through C5aR receptor signaling also recruits and activates monocytes, macrophages, and neutrophils (Roumenina, 2015).

The second activation route is via the lectin pathway (LP). This pathway is triggered by the binding of mannan-binding lectins (MBL), collectin and ficolins to a bacterial/fungal membrane that express pathogen-associated molecular patterns (PAMPs) such as lipoteichoic acid and lipopolysaccharides (Gram-positive bacteria and Gram-negative bacteria, respectively), or β-glucan (fungi). Binding of MBL or ficolins to the pathogenic surface activates the MBL-associated serine proteases (MASP-1, MASP-2, and MASP-3). MASP2 then cleaves C4 and C2 to generate C4bC2a (C3 convertase) in order to initiate the terminal complement cascade (Fujita, 2002). MBL acts as an opsonin signal for immune cells bearing complement or lectin receptors (Takahashi and Ezekowitz, 2005).

The third pathway for activation is by the alternative pathway (AP), which is constitutively active in the host at low levels. Spontaneous low-level hydrolysis of a thioester bond of C3 forms C3(H_2_O), which functions analogous to C3b. C3(H_2_O) further binds to complement factor B (CFB), that is ultimately cleaved by serine protease factor D (CFD), generating a different C3 convertase (C3(H_2_O)Bb) characteristic of the alternative pathway. This C3 convertase complex, similarly to the classical C3 convertase, cleaves C3 to C3a and C3b fragments. Formation of C3 convertase leads to the assembly of the C5 convertase (C3bBb3b) initiating the assembly of the MAC complex on the surface of foreign cells, similar to that of the CP (Dunkelberger and Song, 2009; Ricklin et al., 2010; Merle et al., 2015).

To avoid the destruction of self-components, complement activation needs to be tightly regulated and must be confined to the surfaces of pathogens or dying cells. Complement regulatory proteins serve as crucial factors to regulate complement activation at two stages: at the level of convertases (by cleaving the C3 and C5 convertase components), and during MAC assembly. Some known complement inhibitors such as decay-accelerating factor (DAF; CD55), CR1, Factor H (FH), C4-binding protein (C4BP), and vitronectin, are crucial for restricting complement activation. The mechanism of action of these inhibitors has been summarized in previous reviews (Dunkelberger and Song, 2009; Zipfel and Skerka, 2009). Complement protein factor H, C3b, and iC3b also modulate the neutrophil extracellular trap (NET) released from neutrophils (de Bont et al., 2018).

## Complement Cascade Initiation and Its Anti-*Candida* Effects

Several of the fungal cell wall components (chitin, glucans, and mannans) are recognized by host innate immune components, including complement proteins. In the followings we shortly discuss how each pathway may be involved in anti-*Candida* immune responses based on the currently available information, followed by discussing the role of certain complement proteins (C3 and C5), the terminal clearance system and finally a complement regulator (FH) in *Candida* clearance.

To characterize the chitin-induced complement pathway, neutralizing antibodies against factor B and C1q were used to inhibit the alternative and classical complement pathways. This revealed that antibodies against complement factor B but not against C1q inhibit the cascade induced by purified chitin, suggesting that mainly the AP is activated by this cell wall component (Roy et al., 2013).

The *C. albicans* cell wall component glucan is another potent activator of the AP. Incubation of purified alternative pathway proteins with glucan-displaying *C. albicans* cells effectively initiates the AP (Boxx et al., 2010). Boxx et al. also revealed the importance of anti-mannan antibodies in the ingestion of the mannan-displaying fungal cells and complement proteins in the uptake of glucan-displaying cells by polymorphonuclear leukocytes (PMNs) (Boxx et al., 2010).

MBLs recognize pathogens through their carbohydrate recognition domains (Takahashi and Ezekowitz, 2005; Auriti et al., 2017). MBLs efficiently bind to *C. albicans* mannose and N-acetylglucosamine molecules to activate the lectin pathway (Van Asbeck et al., 2008). Binding of lectins to the *C. albicans* cell wall inhibits growth independent of complement activation (Ip and Lau, 2004).

Deposition of C3 fragments on *C. albicans* activates the complement cascade and enhances opsonophagocytosis by PMNs (Van Asbeck et al., 2008). Mice lacking C3 (C57BL/6 C3^−/−^) are highly susceptible to fungal infections (Tsoni et al., 2009). Another study showed that co-incubation of chitin with human sera or its intratracheal injection in mice induces C3a production (Roy et al., 2013).

B cells are also activated (through B cell receptor complex assembly) upon binding of CR2 (CD21) with C3d-opsonized yeasts on their surface. This assembly not only lowers the activation threshold but also stimulates the production of antibodies via a complement-dependent process (Lyubchenko et al., 2005; Carroll and Isenman, 2012). Affinity of complement proteins also varies for the various cell wall components of the fungus. For instance, complement factors C3b/C3d are more rapidly deposited on β-1,6-glucan compared to β-1,3-glucan. In the same study, unlike β-1,3-glucan, β-1,6-glucan was shown to enhance neutrophil activation, through increased ROS production and uptake, suggesting that deposition of C3d/C3d to β-1,6-glucan on the surface of *C. albicans* could also promote anti-*Candida* effects (Rubin-Bejerano et al., 2007).

C5 convertase cleaves C5, a terminal component of the complement cascade into C5a and C5b. Mice lacking a functional copy of C5 are susceptible to invasive *C. albicans* infection (Mullick et al., 2004). Previously it has been shown that during *C. albicans* infection, C5a activates human monocytes and also induces the production of pro-inflammatory cytokines IL-1β and IL-6 (Yan and Gao, 2012). C5a enhances the *C. albicans*-induced inflammatory response from monocytes through C5a-C5aR signaling, that implies the importance of anaphylatoxins against candidiasis (Cheng et al., 2012). Furthermore, C5a enhances the expression of CR3 (CD11b) on PMNs and C5a-C5aR signaling is also required for neutrophils to migrate to fungal cells (Hünniger et al., 2015; Sun et al., 2015).

Previous studies also revealed a direct anti-*Candida* effect of the terminal complement system. According to Lukasser-Vogl et al. (2000) the presence of opsonized *C. albicans* cells markedly induced the release of C6 and C7 proteins from PMNs, but not that of C8 and C9, suggesting an enhanced assembly of the initial membrane attack complex (Lukasser-Vogl et al., 2000). Another study revealed that the presence of C6/C7 proteins in normal human serum reduced growth, Sap-release and adhesion capabilities of *Candida* cells when compared to C6/C7-depleted conditions. Increased phagocytosis was also detected, suggesting the terminal complex's active inclusion in the augmentation of anti-*Candida* effects (Triebel et al., 2003).

Regarding complement regulators, as mentioned above, complement factor H modulates NET formation. NETs, which consist of chromatin fibers, proteolytic enzymes, and host defense proteins, are able to kill *C. albicans* cells (Urban et al., 2006). FH also acts as a bridge between *C. albicans* and CR3, that further enhances pathogen elimination (Losse et al., 2010).

## Regulation of the Host Complement Cascade by *Candida*

### Recruitment of Complement Regulators on the Cell Surface

**Figure 2:**
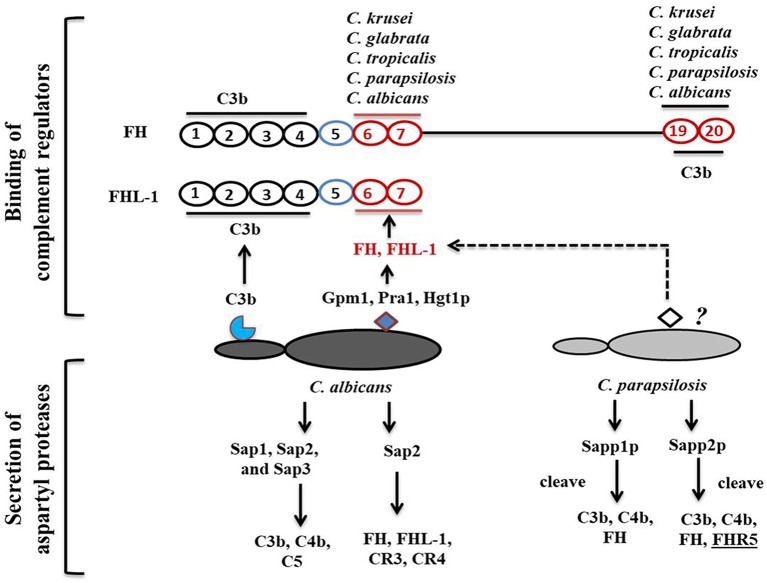
*Candida*-mediated host complement cascade regulation. *C. albicans* regulates the complement attack by two mechanisms: recruiting complement regulators on its surface or secreting aspartyl proteases to cleave complement proteins. *C. albicans* cell surface proteins bind to C3b and CCP domains of complement regulators. *C. albicans* and *C. parapsilosis* can also secrete aspartyl proteases to cleave human complement proteins.

According to Meri et al., besides *C. albicans*, NAC species such as *C. glabrata, C. parapsilosis, C. lusitanae* and *C. tropicalis* also bind complement proteins (Figure 2) (Meri et al., 2002). Activation of the host complement cascade by sensing *Candida* inhibits the growth or facilitates the killing of yeast cells by opsonization. Previous studies suggested that *C. albicans* escapes complement attack by two possible routes: either by recruiting complement regulators [Factor H, FH-like protein (FHL-1), C4BP, plasminogen] on their surface (Table 1) or by degrading complement proteins by proteases (Figure 1) (Meri et al., 2002, 2004; Gropp et al., 2009; Losse et al., 2010; Luo et al., 2013b). Attached to the *C. albicans* cell surface, complement regulator proteins retain their function, and let the fungus regulate and avoid the complement attack (Luo et al., 2013b). The *C. albicans* cell wall-associated proteins Phosphoglycerate mutase (Gpm1p), Glyceraldehyde-3-phosphate dehydrogenase (Gapdh/Gpd), and pH regulated antigen 1 (Pra1p) are confirmed to have a strong binding affinity to complement regulators.

**Table 1:** *C. albicans* cell surface associated proteins important for binding with host complement proteins.

***C. albicans* protein**	**Binds with complement protein**	**References**
Phosphoglycerate mutase (Gpm1p/GPM1)	Factor H, FHL-1, and plasminogen	(Poltermann et al., 2007; Lopez et al., 2014)
pH-regulated antigen 1 (Pra1)	C4BP, C3	(Luo et al., 2010, 2011, 2018)
Translation elongation factor 1 (Tef1p)	FH, C4BP, C3d, C3dg	(Lopez, 2013)
Glyceraldehyde-3-phosphate dehydrogenase (Gapdh)	FH and FHL-1,	(Luo et al., 2013a)
High-affinity glucose transporter 1(CaHgt1p)	FH and C4BP	(Lesiak-Markowicz et al., 2011; Kenno et al., 2019)

Phosphoglycerate mutase (Gpm1p), a cytoplasmic protein, is involved in glycolysis and converts 3-phosphoglyerate to 2- phosphoglycerate (Lopez et al., 2014). The *C. albicans gpm1* deletion mutant has reduced binding of FH and plasminogen compared to the wild-type strain. Attached to *C. albicans* gpm1p, FH and plasminogen remain functional and retain their protease activity (Poltermann et al., 2007). FH serves as a cofactor of factor I (FI). FI, a serine protease, cleaves C3b, and thus inactivates the complement alternative pathway. Surprisingly, NAC members such a *C. krusei, C. glabrata, C. parapsilosis*, and *C. tropicalis* also bind FH and FHL-1 with similar affinity to *C. albicans*, indicating that NAC species could also evade the complement mediated anti-*Candida* host immune response (Meri et al., 2004). In addition to FH on the cell surface of *C. albicans*, gpm1p binds to FHL-1. FH, which consists of 20 complement control protein (CCP) domains, binds to *C. albicans* through its CCP6-7 domain and C terminus CCP19-20 domain (Meri et al., 2002). Similar to FH, CCP-6-7 of FHL-1 can also bind to *C. albicans* (Figure 2). Binding of FH and FHL-1 with NAC members also occurs; however, their binding sites on NAC cell surface are not well-known (Figure 2).

Glyceraldehyde-3-phosphate dehydrogenase (Gapdh/Gpd), which is present on the *C. albicans* cell surface, also appears to bind FH and FHL-1 (Luo et al., 2013a). On the *C. albicans* surface, FH and FHL-1 attached with Gpd retain their complement regulatory activity, thereby inhibiting complement activation (Luo et al., 2013a). Additionally, *C. albicans* binds with plasminogen *via* Gpd2. Surface-bound plasminogen contributes to complement inhibition and degradation of extracellular matrices to help *C. albicans* in tissue invasion (Luo et al., 2013a). pH regulated antigen 1 (Pra1p) is either secreted from *C. albicans* or is cell wall-associated. Hyphae associated Pra1p is highly glycosylated and induces a strong immune response (Marcil et al., 2008; Soloviev et al., 2011; Bergfeld et al., 2017). Pra1p also binds with FH and FH-related protein1 (FHR-1) (Luo et al., 2009). As it is bound to the cell surface of *C. albicans*, Pra1p helps in plasminogen-mediated complement evasion and extra-cellular matrix interaction and/or degradation (Luo et al., 2009). As a secreted protein, it regulates complement activation by binding complement proteins. Pra1 binds to host C3 and blocks the conversion of C3 to C3a and C3b, leading to the inhibition of the complement cascade (Luo et al., 2010). It also helps *C. albicans* evade the complement attack through binding to C4bp, another classical pathway inhibitor. The same study also revealed that *pra1* deletion mutants have a significant but not a complete reduction in C4bp binding. This suggests that other C4bp binding proteins might be present on the cell surface of this species (Luo et al., 2010).

In case of certain pathogens (e.g., parasites and viruses) the “Trojan horse” principle has been previously described, meaning that the pathogen initiates its own uptake by host cells via complement proteins or complement receptors as an alternative immune evasion strategy. Such an event has also been associated with a milder host response, compared to the response followed by a normal opsonization process, enabling intracellular survival and avoiding the hostile extracellular niche (Würzner and Zipfel, 2004). To date, it is still unclear whether *C. albicans* uses a Trojan horse-like mechanism for host cell invasion, let alone if such an event would take place with the help of host complement receptors (Swidergall, 2019). Molecular mimicry of host complement proteins is another potential route of host response evasion. Previously, the presence of human CR3-like proteins was described on the surface of *Candida* cells with C3 binding affinity (Edwards et al., 1986). CR-like molecules of the fungus were also shown to be required for iron acquisition from complement coated red blood cells (Moors et al., 1992). Besides binding C3 in a non-opsonizing manner, CR3-like proteins also enhance host adhesion and invasion (Gustafson et al., 1991).

*C. albicans* also possesses an αvβ3 integrin-like protein similar to the vertebrate αvβ3 integrin receptor (Hostetter, 1999). This protein recruits vitronectin, a terminal complement pathway inhibitor on the surface of this fungus, thereby inhibiting MAC formation (Spreghini et al., 1999).

### Degradation of Complement Proteins

Aspartyl proteases share a common catalytic apparatus, and have a conserved “Asp-Gly-Thr” sequence at their active site. The number of aspartyl acid protease (Sap) encoding genes varies among *Candida* species, as *C. albicans* possesses 10 known *SAP* genes, grouped into 6 subfamilies (*SAP1-3, SAP4-6, SAP7, SAP8, SAP9*, and *SAP10*), while in *C. tropicalis* there is 1 subfamily of four genes (*SAPT1*–*SAPT4*) and *C. parapsilosis* has three genes (*SAPP1–SAPP3*) that have been identified and functionally characterized (Pichová et al., 2001; Naglik et al., 2003). Most studies related to pathogenic *Candida* species are mainly centered on strains either isolated from the oral cavity, vaginal lumen or immunocompromised patients in ICUs. Interestingly, activities of *SAP*s were reported to be variable among these isolates. For instance, strains derived from HIV patients with oral candidiasis or with vaginitis were shown to secrete aspartyl proteases at higher quantities compared to asymptomatic carriers (De Bernardis et al., 1992, 1996). *C. parapsilosis* isolates from skin display higher Sap activity *in vitro* compared to the blood isolates (De Bernardis et al., 1999; Trofa et al., 2008). Research supports the notion of a strong correlation between expansions of *SAP* gene family with pathogenicity of *Candida* species.

Within a host, fungi are able to regulate the complement attack using secreted aspartyl proteases. A previous study showed that Saps, specially Sap1, Sap2, and Sap3 secreted by *C. albicans* cleave C3b, C4b, and C5 proteins, and also block MAC assembly (Gropp et al., 2009). Other Saps, such as Sap9, are not able to cleave complement proteins, only antimicrobial peptides such as histatin5 (Gropp et al., 2009). Sap2 of *C. albicans* also cleaves FH, and FH binding complement receptors CR3 and CR4 on macrophages (Svoboda et al., 2015). *C. albicans* aspartyl proteases also efficiently cleave most immunoglobulins including IgG (Fc portion) and IgA, which are important for complement activation.

Recently, we have shown that secreted aspartyl proteases in *C. parapsilosis*, especially Sapp1p and Sapp2p can also cleave C4b, C3b, and FH, however their specificity and cleavage capacity differs. Sapp1p has a higher cleavage capacity against C3b compared to Sapp2p, whereas only Sapp2p but not Sapp1p cleaves FHR-5 (Singh et al., 2019).

In addition to complement proteins, aspartyl proteases of *C. albicans* can efficiently hydrolyze, cleave or activate other host defense proteins, including salivary lactoferrin, lactoperoxidase, immunoglobulins, cathepsin D, IL-1β, human big endothelin-1, α2-macroglobulin, etc. (Germaine et al., 1978; Ruchel, 1984; Kaminishi et al., 1990).

## Conclusion

Neonates, the elderly, and patients with an acquired or inherited underlying immunocompromised status are the most vulnerable to invasive candidiasis. To effectively combat *Candida* infections, the complement system is of particular importance due to its direct interaction with fungal cells and, consequently, an effective innate or adaptive immune response. Such responses include the activation of macrophages, neutrophils and dendritic cells or B cells as a result of pathogen opsonization. Chemotactic recruitment of immune cells at the site of infection is mediated by anaphylotoxins (C3a, C5a), concomitantly resulting in enhanced internalization, oxidative burst and the secretion of proinflammatory cytokines by activating the complement receptors on immune cells are common complement-mediated defense mechanisms.

Highly virulent *Candida* species have evolved mechanisms to evade the host's complement attack. These processes include the binding of complement regulators on their surface and secretion of proteases to degrade complement cascade initiating components. Therefore, insights into the multifaceted interactions between human complement proteins and pathogenic *Candida* species may allow us to develop promising approaches for therapeutic strategies targeting complement proteins involved in the pathogenesis of *Candida* infections.

## Author Contributions

DS wrote the manuscript and prepared the figures. The manuscript was reviewed by RT and AG. All authors read the final version and approved the last version.

### Conflict of Interest

The authors declare that the research was conducted in the absence of any commercial or financial relationships that could be construed as a potential conflict of interest.
